# Palliative Care for Stroke Patients and Their Families: Barriers for Implementation

**DOI:** 10.3389/fneur.2019.00164

**Published:** 2019-03-06

**Authors:** Tobias Steigleder, Rainer Kollmar, Christoph Ostgathe

**Affiliations:** ^1^Department of Palliative Care, University Hospital Erlangen-Nuremberg, Erlangen, Germany; ^2^Department of Neurology, University Hospital Erlangen-Nuremberg, Erlangen, Germany; ^3^Department of Neurology and Neurointensive Care, Darmstadt Academic Hospital, Darmstadt, Germany

**Keywords:** stroke, palliative care, palliative care needs, family, next-of-kin, caregiver burden, early integration, palliative care indication

## Abstract

Stroke is a leading cause of death, disability and is a symptom burden worldwide. It impacts patients and their families in various ways, including physical, emotional, social, and spiritual aspects. As stroke is potentially lethal and causes severe symptom burden, a palliative care (PC) approach is indicated in accordance with the definition of PC published by the WHO in 2002. Stroke patients can benefit from a structured approach to palliative care needs (PCN) and the amelioration of symptom burden. Stroke outcome is uncertain and outlook may change rapidly. Regarding these challenges, core competencies of PC include the critical appraisal of various treatment options, and openly and respectfully discussing therapeutic goals with patients, families, and caregivers. Nevertheless, PC in stroke has to date mainly been restricted to short care periods for dying patients after life-limiting complications. There is currently no integrated concept for PC in stroke care addressing the appropriate moment to initiate PC for stroke patients, and the question of how to screen for symptoms remains unanswered. Therefore, PC for stroke patients is often perceived as a stopgap in cases of unfavorable prognosis and very short survival times. In contrast, PC can provide much more for stroke patients and support a holistic approach, improve quality of life and ensure treatment according to the patient's wishes and values. In this short review we identify key aspects of PC in stroke care and current barriers to implementation. Additionally, we provide insights into our approach to PC in stroke care.

## Introduction

Stroke has all the characteristics of a disease consistent with the mandate of palliative care (PC) as defined by the WHO in 2002 (1): (a) PC addresses patients with life-threatening diseases, regardless of individual prognosis; stroke shows a 1-year mortality of 30–40%, it is the second leading cause of death worldwide. Its global burden of disease is continuously rising (2, 3); (b) PC addresses quality of life (QoL) as primary outcome parameter. QoL is severely impaired following stroke. There is evidence that stroke patients and families suffer from anxiety and decreased self-worth; they feel that they lack information, have difficulty sharing feelings and emotions. This was the result of an assessment six weeks after stroke. After six months and one year, respectively anxiety remained prominent for both patients and next-of-kin (4); (c) PC assesses and ameliorates symptom burden (SB) in various dimensions. SB is severe in stroke patients, comprising somatic, social, psychological, and spiritual aspects (5, 6). In a neuro-critical care unit setting with mostly stroke patients, two thirds of patients and families reported PC needs (PCN) (7).

Integration of PC in treatment of stroke has been demanded repeatedly and data points to its beneficial effect (4, 8). PC can reduce SB after stroke (9) and shorten the length of hospital stay (10). PC correlated with longer time of survival after acute stroke (11) in parallel to its effects in cancer and dyspnoae patients (12–14).

Integration of PC in stroke care has been required by various professional societies (15, 16). In consequence, Creutzfeldt and Holloway demanded that stroke specialists must be able to deliver primary palliative care, comprising (1) patient- and family-centered care, (2) prognosis estimation, (3) development of appropriate goals of care, (4) awareness of end-of-life implications for common stroke decisions, (5) assessment and management of symptoms, (6) experience with palliative treatments at the end of life, (7) care coordination, including referral to PC or hospice, (8) fostering personal growth for patient and family, (9) ensuring the availability of bereavement resources if death is anticipated and (10) participation in quality improvement and research (15). Still, less than one in 15 stroke patients receive PC, whereas more than half of stroke patients die within 12 months or remain severely impaired with unknown SB and PCN (8, 17). Despite the need of a comprehensive approach, Ackroyd and Nair state that PC is still mainly integrated to co-manage the last days of life and to help with the determination of treatment goals (18). We identified crucial topics for PC in stroke and barriers to its timely and adequate implementation.

### The Right Time for Palliative Care Involvement

Laymen and health care specialists alike may view the term palliative care (PC) as related to giving up curative or life-prolonging treatment and essentially on the patients themselves (19, 20). Accordingly, stroke specialists often consider PC applicable only in the phase of dying (4) or in the certain case of a poor prognosis (11), and may even equate PC with a decision against any treatment at all (21). In a qualitative study with 33 health care professionals specialized in stroke, only one participant understood that prognostic uncertainty may persist after the introduction of palliative care (4).

**Figure 1:**
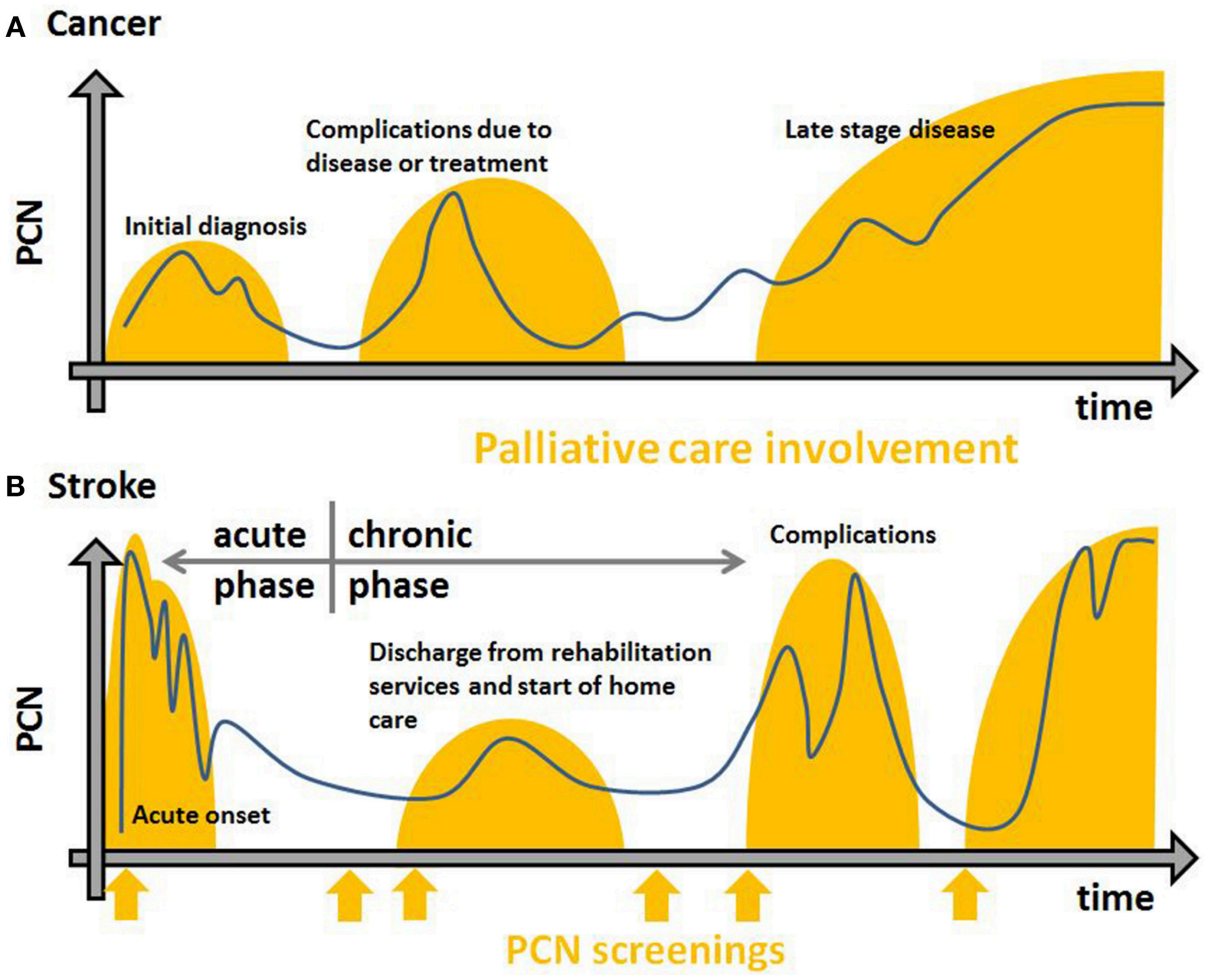
Exemplary trajectories of chronic diseases **(A)**, e.g., cancer, heart failure, moto-neuron disease, Parkinson's disease and acute diseases **(B)**, e.g., stroke in terms of PCN (y-axis) over time (x-axis). The blue lines are possible courses of SB over time. Orange orbs show PC involvement. Initiation in a timely fashion to achieve best results is based on regular standardized screenings for PCN (orange arrows).

Indication for PC changes over the course of a disease (22). In cancer patients, PC is part of a comprehensive treatment concept, which is implemented in parallel with anticancer therapy as an integrated approach (23). If curative treatment has been unsuccessful over the course of the disease, patients will eventually no longer benefit from anticancer therapy and PC will be offered exclusively (22). Incurable cancer progresses gradually and impacts overall health. In stroke, the course of the disease is different: an acute onset—if not instantly lethal—is followed in most cases by a chronic rehabilitation period (Figure 1). In acute stroke, PC involvement is often initiated to support care of the dying (18), but in the chronic stage of stroke, PCN may remain high, increase, or reappear (9). For post stroke patients, treatment is focused on rehabilitation, secondary prevention, and care management, but PC needs (PCN) are neither regularly screened for nor routinely treated (9, 24). Especially patients with cognitive and communicative impairments found it difficult to get access to services and equipment and often felt abandoned. This impression was reinforced once health care professionals decided that they had reached a stable plateau and curative and rehabilitation offers were withdrawn (4). Conversely, many doctors will refrain from offering PC to address mental, social, and spiritual SB at this stage, as they fear such measures would be understood as a signal of abandonment (11).

Structured screening programs can help to overcome this barrier and Creutzfeldt has suggested the use of a “Palliative care needs checklist” to screen for PCN, as seen below:

- Does this patient have pain or distressing symptoms?
- Does the patient and/or their family need social support or help with coping?
- Do we need to readdress goals of care or adjust treatment according to patient-centered goals?
- *What needs to be done today?* (25).

### Therapeutic Goals and Communication

Stroke may lead to severe loss of function. Whether loss of function causes “unacceptable” circumstances of living differs subjectively. In a mixed method investigation, some stroke patients with rather severe disabilities accepted their disability and some with less severe disabilities felt discontented up to the point to claim that death would have been preferable (4). Interviews with stroke patients, next-of-kin and formal caregivers revealed that thoughts of death were common, but were not addressed with formal caregivers, who hope for good recovery even in cases with death as a possible outcome. Staff admitted to be overoptimistic in order to motivate patients, especially when encouraging them to participate in physical therapy (4).

Communicating therapeutic goals and possible outcomes truthfully can help to avoid vast discrepancies between experiences and expectations (26). The further patients' and families' experiences deviate from communicated goals and expectancies, the lower their satisfaction and overall QoL will be (27–29). Uncertainty of prognosis is a main stressor for families of stroke patients (30). Informal caregivers often feel that advance planning for both recovery and deterioration would help to address this issue (4).

Defining therapeutic goals after acute stroke faces various challenges. Decisions must be based both on medical evidence and on patients' personal preferences (30). Stroke occurs as a sudden and unexpected life event often with severe impact on all aspects of life including cognition and communication. Living wills can be helpful here, but they are not always available. Therefore, determining the will of the patient, when communication and/or decision-making capacities have been lost, is particularly challenging and often relies on narratives by proxies. Limitations in experience, resources, and self-perceived qualification present additional barriers for health care professionals to successfully elicit the patient's point of view (11, 15, 31). Families describing their loved ones are likely to remember them as having been healthier and more autonomous than they actually were (recall bias) (30). In consequence, treatment outcomes may appear unfavorable although they are in accordance with the patient's life as it was. Also, perception of patients on favorable outcomes may change and we know QoL of patients to improve in the course of disabling diseases (32–34) and to be better than the QoL healthy participants expect when imagining to experience comparable circumstances (35). For example, most healthy persons said they would decide against hemicraniectomy facing the odds of disablement in case they had a severe stroke (36), whereas most people having been treated by hemicraniectomy after stroke would make the same choice again being given the same situation (37). Weighing values of future outcomes is directly influenced by perception of loss and gain based on the current status. Kahneman coined the term “losses loom larger than gains” (38), describing that a loss is felt more intensely than a possible gain, as is the case with imagining a new life situation when the focus lies on loss of function (speech, mobility, autonomy) in contrast to perceived values (gaining rehabilitation, social participation, and life) (30, 39). This psychological phenomenon has to be addressed by health care specialists when discussing treatment options in stroke.

PC involvement is often initiated to elicit goals of care together with the patient, the family and the stroke care team and also to support advance care planning. In a large retrospective series, PC involvement was found to triple advance directives (AD) while standard stroke care achieved an increase by 50% (7). In addition, AD exceeds mere planning for sudden deterioration, as it also serves to communicate therapeutic alternatives as well as to encourage and systematize quality care for severely ill patients (16). As therapeutic contacts will become less frequent after discharge from clinics and rehabilitation facilities, stroke patients can profit from AD through improvement of long term care.

### Identification of Palliative Care Needs

Palliative care needs (PCN) are common and substantial after stroke (15, 24, 25, 40, 41). Both patients and families report lower health-related quality of life (HRQOL) after stroke (9, 15). Certain populations are especially at risk for inadequate amelioration of SB, including very young and old patients as well as patients with impaired communication skills (42).

Data points to the fact that short term PCN in the last days of life are different from PCN among stroke survivors, with only few studies shedding light on long-term SB.

Several studies have investigated PCN in stroke patients with a focus on the last days of life. Here, most common somatic symptoms are dyspnoea (30%), pain (25–30%, mostly central post-stroke pain, hemiplegic shoulder pain, and spasticity induced pain), xerostomia (20%), constipation (20%), sadness (35–50%), anxiety (25%), and fatigue (50%) (6, 9, 43, 44). Although burdensome symptoms are sporadically recognized by stroke specialists, there is a lack of awareness and attention. Symptoms are attributed to stroke as part of the natural course rather than being viewed as treatable distress (43, 45, 46).

A structured approach is needed to identify PCN after stroke, but no appropriate tool has been developed so far (6). The Sheffield Profile for Assessment and Referral to Care (SPARC) has been proposed as a screening tool to identify patients who may profit from PC (40) and was successful in regards to acceptance and feasibility in a roll-out trial with 135 patients with various diseases (47). Whereas, SPARC covers PCN extensively, it contains 45 items and is challenging for patients with cognitive impairment. It has not been validated in patients with communication impairment, although elderly patients and those with impaired communication skills are in increased danger of untreated SB after stroke (42). The Palliative outcome scale (POS) is a validated and multidimensional assessment tool. POS comprises 11 items and addresses SB in somatic, psychological, social, and spiritual dimensions. Additionally, POS allows for multicenter comparison and thereby supports research endeavors. POS is widely accepted and has been adapted for multiple sclerosis and Parkinson's disease, but not for stroke.

### Place of Death

Stroke causes severe restrictions in terms of autonomy and self-care. The level of professional care after stroke is high and many people remain in hospitals and rehabilitation clinics for a considerable length of time (48). When discharged, many are transferred to nursing homes, even though most persons wish their homes to be the place of care and their place of death (49). Within 1 year after stroke, about two thirds of patients die in a hospital and only one in ten dies at home. Death in hospices was not specifically recorded (50). Only 10–12% of all deaths were found to be unexpected. For only 6% of patients who died in a hospital, their place of death corresponded to their explicit will. In contrast, 39% of patients who died in nursing homes and 78% of patients who died at home had expressed wishes to die there (50).

### Caregiver Burden

A mixed-methods study showed that stroke is a major life crisis for patients as well as for next-of-kin (4). Stroke has a severe and sudden effect on physical, behavioral, and psychological functions, impacting all social interactions (51). Family members were unsure whether they were “doing the right thing” and were confused by health care professionals who expressed controversial narratives of good recovery vs. accounts of disability and death (4). Uncertainty of prognosis and possibility of a second stroke contributed to the strain especially strongly (30). Next-of-kin reported severe burdens on social structures (21, 52) and anxiety, partially due to lack of information, and emotional distress remained severe up to 1 year after the stroke (4).

Additionally, as most stroke patients die in a hospital (50), next-of-kin may be restricted in spending time with the patient through hospital regulations and logistic challenges, which increases caregiver burden. For patients who die in palliative and hospice care, next-of-kin report less posttraumatic stress disorder and facilitated grieving (53) as well as higher satisfaction with end-of-life care (54).

For next-of-kin in a mixed population, including mainly cancer patients, the burden is effectively alleviated by involvement of PC services (55). Especially in a multidimensional approach, PC improves quality of care significantly; main topics of significant improvement are: religious/spiritual beliefs, adequate support in dealing with one's own feelings, feelings after the possibility of death has been addressed, referral to psychosocial support for family, assessment of emotional/spiritual needs, support of the family's self-efficacy, and mild to strong confidence within families to know what to expect as well as what to do when the patient would die. Data shows that burden and need of support of next-of-kin increases if patients' cognitive functions are impaired (56–58). Uncertainty of outcome leads to a rise in burden for next-of-kin and patients (59). Both factors of increased burden for next-of-kin are highly prevalent in stroke patients. Although informal care giver burden is of great significance for both the individual (60) and society (61, 62), screening tools and instruments to assess informal caregiver burden in stroke are needed (63) as well as systematic research into suitable interventions (64).

## Own Experiences and Conclusion

The appropriate point-of-time to integrate PC is a main challenge in implementing PC, especially for stroke patients and their families. PC has its origins in end-of-life care for cancer patients, which was reflected in the WHO technical report series of 1990 (65). Much has changed in favor of patients. Today, PC is understood as an integrated service which works in conjunction with other medical specialties in order to improve QoL and ameliorate SB regardless of prognosis in case of any life-threatening disease (1, 23, 66). By systematic and early integration of PC several beneficial effects have been found, e.g., reduction of SB and depression, increase of QoL, satisfaction of next-of-kin, and likelihood of survival in cancer patients (12–14). PC has been of increasing importance in neurology (20, 67), but it integrates more easily in some subspecialties, e.g., moto-neuron diseases, Parkinson's disease, and multiple sclerosis than in others like stroke (30) as the clinical course of stroke is fundamentally different from that of the aforementioned (Figure 1).

The main barrier of integration of PC in stroke is the obsolete idea of PC as being invariably joined with both definite and poor prognoses and automatic withdrawal of stroke care. Even health professionals still confuse these aspects (68). This is paralleled in the case of other vascular diseases like heart failure. In congestive heart failure, the foremost barrier for integration of PC the incorrect perception of PC being prognosis-dependent and requiring suspension of life-prolonging treatment (19). A suggestion on how to move from “prognostic paralysis to active total care” is to focus on patients who “reasonably might die” rather than patients who “will die in the next six months” (69, 70), as is also reflected in the surprise question “Would I be surprised if my patient were to die in the next 12 months?” (71). If the answer to the surprise question is “no,” a detailed PCN screening is necessary. Whether the time span of 12 months that has been validated in cancer patients can be paralleled to the course of disease in neurological non-cancer patients has not yet been researched.

We endorse Creutzfeldt's proposition of the PCN checklist (25). However, as it has neither been standardized nor validated, it may still be difficult for a stroke specialist to apply the PCN checklist in practice. A standardized and structured approach will be necessary to screen for and identify PCN.

Integrated pathways of care have proven to increase quality of care for stroke patients and for patients in hospice and palliative care, respectively (72–74). Still, an integrated PC approach has not yet been implemented in stroke care. Beyond initial and acute evaluation, regular assessments are crucial to ensure the identification of stroke patients who develop PCN later in the course of their disease due to complications, deterioration, and increasing distress in informal caregivers (21). Existing PC screening instruments, which are mostly derived from research on cancer patients' PCN, do not reflect specific SB of patients with chronic illnesses. For some neurological entities, adaptations of such screening tools have been developed, e.g., POS for Parkinson's disease and multiple sclerosis.

We have implemented a structured approach for stroke patients, based on a questionnaire which is sent to patients or next-of-kin within 6 months after discharge. The questionnaire constitutes a self-assessment tool which screens for PCN in four domains (physical, mental, social, spiritual) and has been validated for PC patients (75) and neurological outcome measures such as modified Rankin Scale and Barthel index. In addition, we address spasticity-associated pain and discomfort as specific and treatable symptoms. We use 20 pictures showing spastic postures of extremities and 16 questions aiming at symptoms related to post-stroke spasticity. The questionnaire is designed for self-assessment or assessment by proxy and the evaluation of its content is based on cumulative scores (76, 77).

Our approach aims at the first months after discharge with a focus on post-stroke spasticity. To ensure a holistic approach to PCN after stroke, future research is urgently needed to identify and quantify stroke-specific parameters and develop appropriate intervals for PCN screening in the late phase of stroke.

In order to deliver PC to stroke patients and their families, more needs to be known of stroke-specific PCN. Stroke-specific PCN may be treated differently from similar symptoms in cancer care. Post-stroke pain, for example, is a common symptom, but management stemming from cancer pain might not be the most efficient way to ameliorate this pain. Opioids may reduce alertness and worsen constipation (78), whereas focal interventions like injections with botulinum toxin may help more in case of spasticity with significantly less adverse side effects (79).

PC addresses both the patient and the family. Next-of-kin are severely burdened by a rapidly changing situation in life, changes of the patient's functional and psychological status, and responsibilities in home care. Cancer-focused research showed that targeted interventions to increase QoL for the patient do not automatically lead to an increase of QoL of their family (80, 81). Therefore, it is necessary to develop this tailored support for caregivers (82).

Defining appropriate therapeutic goals and discussing alternatives are necessary steps in all phases of stroke. Next-of-kin are fraught with uncertainty by contradictory narratives simultaneously aiming at a good outcome and describing catastrophic development in dual presentation (4, 83). Uncertainty is a main factor of distress (30), but may be ameliorated decisively by addressing it openly and engaging in a transparent and meaningful dialogue about possible outcomes and therapeutic goals (4, 27, 38). To openly discuss the uncertainty of health outcomes with patients, families, and formal caregivers is a key competence in PC (70). AD may yield an appropriate platform to initiate this discourse with the added benefit to ensure patients' wishes in further course of the disease. To achieve this goal, further education of health care specialists on communication skills is needed. An open discussion on therapeutic goals may foster a trusting relation with patients and their families.

In the future, more research and more openness on both sides—palliative care specialists and neurologists—is needed to better understand PCN of stroke patients and their families and to assess how to ameliorate stroke-specific SB. Early detection and tailored interventions may prevent exacerbation of symptoms, reduce the involvement of emergency services and thereby health costs, prolong patients' lives, reduce suffering, increase QoL for patients and families, and allow patients to remain and possibly to die at home in care of their loved ones.

## Author Contributions

TS: concept, first draft, and final version. RK: essential input and formative evaluation. CO: concept, essential input, formative evaluation, and final version.

### Conflict of Interest Statement

The authors declare that the research was conducted in the absence of any commercial or financial relationships that could be construed as a potential conflict of interest.

## Abbreviations

AD: Advance Directives
CSI: Carer Strain Index
DALYs: Disability Adjusted Life Years
EQ5D: Quality of Life Questionnaire
GBD: Global Burden of Disease
HRQOL: Health Related Quality of Life
PC: Palliative Care
PCN: Palliative Care Needs
POS: Palliative Outcome Scale
QoL: Quality of Life
SB: Symptom Burden
SPARC: Sheffield Profile for Assessment and Referral to Care.

## References

[1] Sepúlveda C Marlin A Yoshida T Ullrich A. Palliative care: the World Health Organization's global perspective. J Pain Sympt Manage. (2002) 24:91–6. 10.1016/S0885-3924(02)00440-2 12231124

[2] Feigin VL Norrving B Mensah GA. Global burden of stroke. Circ Res. (2017) 120:439–48. 10.1161/CIRCRESAHA.116.308413 28154096

[3] Collins TC Petersen NJ Menke TJ Souchek J Foster W Ashton CM. Short-term, intermediate-term, and long-term mortality in patients hospitalized for stroke. J Clin Epidemiol. (2003) 56:81–7. 10.1016/S0895-4356(02)00570-X 12589874

[4] Kendall M Cowey E Mead G Barber M McAlpine C Stott DJ. Outcomes, experiences and palliative care in major stroke: a multicentre, mixed-method, longitudinal study. Can Med Assoc J. (2018) 190:E238–46. 10.1503/cmaj.170604 29507155 PMC5837872

[5] Ntlholang O Walsh S Bradley D Harbison J. Identifying palliative care issues in inpatients dying following stroke. Irish J Med Sci. (2016) 185:741–4. 10.1007/s11845-015-1290-9 25851714

[6] Mazzocato C Michel-Nemitz J Anwar D Michel P. The last days of dying stroke patients referred to a palliative care consult team in an acute hospital. Eur J Neurol. (2010) 17:73–7. 10.1111/j.1468-1331.2009.02744.x 19614968

[7] Creutzfeldt CJ Engelberg RA Healey L Cheever CS Becker KJ Holloway RG. Palliative care needs in the neuro-ICU. Crit Care Med. (2015) 43:1677–84. 10.1097/CCM.0000000000001018 25867905 PMC4506250

[8] Williams MT Zimmerman E Barry M Trantum L Dietrich MS Doersam JK. A retrospective review of patients with acute stroke with and without palliative care consultations. Am J Hospice Palliat Med. (2018) 36, 60–4. 10.1177/1049909118787136 29991277

[9] Creutzfeldt CJ Holloway RG Walker M. Symptomatic and palliative care for stroke survivors. J Gen Intern Med. (2012) 27:853–60. 10.1007/s11606-011-1966-4 22258916 PMC3378740

[10] Schloss ER Early palliative care consultation decreases length of stay in severe stroke patients (P1. 211). Neurology. (2017) 88 (16 Suppl.).

[11] Quadri SZ Huynh T Cappelen-Smith C Wijesuriya N Mamun A Beran RG. Reflection on stroke deaths and end-of-life stroke care. Intern Med J. (2018) 48:330–4. 10.1111/imj.13619 28892278

[12] Bakitas MA Tosteson TD Li Z Lyons KD Hull JG Li Z. Early versus delayed initiation of concurrent palliative oncology care: patient outcomes in the ENABLE III randomized controlled trial. J Clin Oncol. (2015) 33: 1438–45. 10.1200/JCO.2014.58.6362 25800768 PMC4404422

[13] Higginson IJ Bausewein C Reilly CC Gao W Gysels M Dzingina M. An integrated palliative and respiratory care service for patients with advanced disease and refractory breathlessness: a randomised controlled trial. Lancet RespirMed. (2014) 2:979–87. 10.1016/S2213-2600(14)70226-7 25465642

[14] Temel JS Greer JA Muzikansky A Gallagher ER Admane S Jackson VA. Early palliative care for patients with metastatic non–small-cell lung cancer. N Engl J Med. (2010) 363:733–42. 10.1056/NEJMoa1000678 20818875

[15] Holloway RG Arnold RM Creutzfeldt CJ Lewis EF Lutz BJ McCann RM. Palliative and end-of-life care in stroke: a statement for healthcare professionals from the American Heart Association/American Stroke Association. Stroke. (2014) 45:1887–916. 10.1161/STR.0000000000000015 24676781

[16] Johnson S Kerridge I Butow PN Tattersall MH. Advance Care Planning: is quality end of life care really that simple? Intern Med J. (2017) 47:390–94. 10.1111/imj.13389 28401724

[17] Singh T Peters SR Tirschwell DL Creutzfeldt CJ. Palliative care for hospitalized patients with stroke: results from the 2010 to 2012 national inpatient sample. Stroke. (2017) 48:2534–40. 10.1161/STROKEAHA.117.016893 28818864 PMC5571885

[18] Ackroyd J Nair A 114 Palliative care and the acute stroke ward: new beginnings? BMJ.

[19] Kavalieratos D Mitchell EM Carey TS Dev S Biddle AK Reeve BB “Not the ‘grim reaper service”’: an assessment of provider knowledge, attitudes, and perceptions regarding palliative care referral barriers in heart failure. J Am Heart Assoc. (2014) 3:e000544 10.1161/JAHA.113.000544 24385453 PMC3959712

[20] Boersma I Miyasaki J Kutner J Kluger B. Palliative care and neurology: time for a paradigm shift. Neurology. (2014) 83:561–7. 10.1212/WNL.0000000000000674 24991027 PMC4142002

[21] Braun LT Grady KL Kutner JS Adler E Berlinger N Boss R. Palliative care and cardiovascular disease and stroke: a policy statement from the American Heart Association/American Stroke Association. Circulation. (2016) 134:e198–225. 10.1161/CIR.0000000000000438 27503067

[22] Ferris FD Bruera E Cherny N Cummings C Currow D Dudgeon D Palliative cancer care a decade later: accomplishments, the need, next steps—from the American Society of Clinical Oncology. J Clin Oncol. (2009) 27:3052–8. 10.1200/JCO.2008.20.1558 19451437

[23] Smith TJ Temin S Alesi ER Abernethy AP Balboni TA Basch EM. American Society of Clinical Oncology provisional clinical opinion: the integration of palliative care into standard oncology care. J Clin Oncol. (2012) 30:880–7. 10.1200/JCO.2011.38.5161 22312101

[24] Stevens T Payne SA Burton C Addington-Hall J Jones A. Palliative care in stroke: a critical review of the literature. Palliat Med. (2007) 21:323–31. 10.1177/0269216307079160 17656409

[25] Creutzfeldt CJ Holloway RG Curtis JR. Palliative care: a core competency for stroke neurologists. Stroke. (2015) 46:2714–9. 10.1161/STROKEAHA.115.008224 26243219 PMC4553237

[26] Carr AJ Gibson B Robinson PG Is quality of life determined by expectations or experience? BMJ. (2001) 322:1240–3. 10.1136/bmj.322.7296.1240 11358783 PMC1120338

[27] Calman KC. Quality of life in cancer patients–an hypothesis. J Med Ethics (1984) 10:124–7. 6334159 10.1136/jme.10.3.124 PMC1374977

[28] Ross CK Frommelt G Hazelwood L Chang RW. The role of expectations in patient satisfaction with medical care. J Health Care Market. (1987) 7:16–26. 10302171

[29] Roberts AH Kewman DG Mercier L Hovell M The power of nonspecific effects in healing: implications for psychosocial and biological treatments. Clin Psychol Rev. (1993). 13:375–91. 10.1016/0272-7358(93)90010-J

[30] Creutzfeldt CJ Holloway RG. Treatment decisions after severe stroke: uncertainty and biases. Stroke. (2012) 43:3405–8. 10.1161/STROKEAHA.112.673376 23150658

[31] Luckett T Spencer L Morton RL Pollock CA Lam L Silvester W. Advance care planning in chronic kidney disease: a survey of current practice in Australia. Nephrology. (2017) 22:139–49. 10.1111/nep.12743 26860214

[32] Headey B Holmström E Wearing A The impact of life events and changes in domain satisfactions on well-being. Soc Indic Res. (1984) 15:203–27. 10.1007/BF00668671

[33] Allison PJ Locker D Feine JS. Quality of life: a dynamic construct. Soc Sci Med. (1997) 45:221–30. 10.1016/S0277-9536(96)00339-5 9225410

[34] Ubel PA Peeters Y Smith D. Abandoning the language of “response shift”: a plea for conceptual clarity in distinguishing scale recalibration from true changes in quality of life. Qual Life Res. (2010) 19:465–71. 10.1007/s11136-010-9592-x 20112000

[35] Albrecht GL Devlieger PJ. The disability paradox: high quality of life against all odds. Soc Sci Med. (1999) 48:977–88. 10.1016/S0277-9536(98)00411-0 10390038

[36] Klein A Kuehner C Schwarz S. Attitudes in the general population towards hemi-craniectomy for middle cerebral artery (MCA) infarction. A population-based survey. Neurocrit Care. (2012) 16:456–61. 10.1007/s12028-012-9677-1 22311231

[37] Creutzfeldt CJ Schubert GB Tirschwell DL Longstreth WT Jr Becker KJ Risk of seizures after malignant MCA stroke and decompressive hemicraniectomy. Am Heart Assoc. (2012) 43.

[38] Kahneman D Tversky A Choices, values, and frames. In: MacLean LC Ziemba WT editors. Handbook of the Fundamentals of Financial Decision Making: Part I. Singapore: World Scientific; WSPC (2013). p. 269–78.

[39] Kahneman D Krueger AB Schkade D Schwarz N Stone AA. Would you be happier if you were richer? A focusing illusion. Science. (2006) 312:1908–10. 10.1126/science.1129688 16809528

[40] Burton CR Payne S Addington-Hall J Jones A. The palliative care needs of acute stroke patients: a prospective study of hospital admissions. Age Ageing. (2010) 39:554–9. 10.1093/ageing/afq077 20647596

[41] Mead GE Cowey E Murray SA Life after stroke–is palliative care relevant? A better understanding of illness trajectories after stroke may help clinicians identify patients for a palliative approach to care. Int J Stroke. (2013) 8:447–8. 10.1111/ijs.12061 23879749

[42] Cristian A Giammarino C Olds M Adams E Moriarty C Ratner S. Safety considerations for patients with communication disorders in rehabilitation medicine settings. Phys Med Rehabil Clin N Am. (2012) 23:343–7. 10.1016/j.pmr.2012.02.017 22537697

[43] Hackett ML Yapa C Parag V Anderson CS. Frequency of depression after stroke: a systematic review of observational studies. Stroke. (2005) 36:1330–40. 10.1161/01.STR.0000165928.19135.35 15879342

[44] Campbell Burton CA Murray J Holmes J Astin F Greenwood D Knapp P. Frequency of anxiety after stroke: a systematic review and meta-analysis of observational studies. Int J Stroke. (2013) 8:545–59. 10.1111/j.1747-4949.2012.00906.x 23013268

[45] Burton CR Payne S. Integrating palliative care within acute stroke services: developing a programme theory of patient and family needs, preferences and staff perspectives. BMC Palliat Care. (2012) 11:22. 10.1186/1472-684X-11-22 23140143 PMC3539873

[46] Dar SK Venigalla H Khan AM Ahmed R Mekala HM Zain H Post stroke depression frequently overlooked, undiagnosed, untreated. Neuropsychiatry. (2017) 7:906–19. 10.4172/Neuropsychiatry.1000296

[47] Hughes P Ahmed N Winslow M Walters SJ Collins K Noble B. Consumer views on a new holistic screening tool for supportive and palliative-care needs: Sheffield Profile for Assessment and Referral for Care (SPARC): a survey of self-help support groups in health care. Health Expect. (2015) 18:562–77. 10.1111/hex.12058 23414548 PMC5060805

[48] Thom T Haase N Rosamond W Howard VJ Rumsfeld J Manolio T. Heart disease and stroke statistics-−2006 update: a report from the American Heart Association Statistics Committee and Stroke Statistics Subcommittee. Circulation. (2006) 113:e85–151. 10.1161/CIRCULATIONAHA.105.171600 16407573

[49] Higginson IJ Sen-Gupta GJ. Place of care in advanced cancer: a qualitative systematic literature review of patient preferences. J Palliat Med. (2000) 3:287–300. 10.1089/jpm.2000.3.287 15859670

[50] Asplund K Lundström S Stegmayr B End of life after stroke: A nationwide study of 42,502 deaths occurring within a year after stroke. Eur Stroke J. (2018) 3:74–81. 10.1177/2396987317736202 PMC6453235 31008338

[51] Haley WE Roth DL Hovater M Clay OJ. Long-term impact of stroke on family caregiver well-being: a population-based case-control study. Neurology. (2015) 84:1323–9. 10.1212/WNL.0000000000001418 25740862 PMC4388745

[52] de Kloet AJ Lambregts SA Berger MA van Markus F Wolterbeek R Vliet Vlieland TP. Family impact of acquired brain injury in children and youth. J Dev Behav Pediatr. (2015) 36:342–51. 10.1097/DBP.0000000000000169 25961902

[53] Wright AA Keating NL Balboni TA Matulonis UA Block SD Prigerson HG. Place of death: correlations with quality of life of patients with cancer and predictors of bereaved caregivers' mental health. J Clin Oncol. (2010) 28:4457. 10.1200/JCO.2009.26.3863 20837950 PMC2988637

[54] Sadler E Hales B Henry B Xiong W Myers J Wynnychuk L. Factors affecting family satisfaction with inpatient end-of-life care. PLoS ONE. (2014) 9:e110860. 10.1371/journal.pone.0110860 25401710 PMC4234251

[55] Gelfman LP Meier DE Morrison RS. Does palliative care improve quality? A survey of bereaved family members. J Pain Sympt Manage. (2008) 36:22–8. 10.1016/j.jpainsymman.2007.09.008 18411019 PMC2527760

[56] Edvardsson T Ahlström G. Being the next of kin of a person with a low-grade glioma. Psycho Oncol. (2008) 17:584–91. 10.1002/pon.1276 17957731

[57] Wideheim AK Edvardsson T Påhlson A Ahlström G. A family's perspective on living with a highly malignant brain tumor. Cancer Nurs. (2002) 25:236–44. 10.1097/00002820-200206000-00012 12040233

[58] Madsen K Poulsen HS. Needs for everyday life support for brain tumour patients' relatives: systematic literature review. Eur J Cancer Care. (2011) 20:33–43. 10.1111/j.1365-2354.2010.01184.x 20477857

[59] Newton C Mateo MA. Uncertainty: strategies for patients with brain tumor and their family. Cancer Nurs. (1994) 17:137–40. 8019997

[60] Adelman RD Tmanova LL Delgado D Dion S Lachs MS. Caregiver burden: a clinical review. JAMA. (2014) 311:1052–60. 10.1001/jama.2014.304 24618967

[61] Nielsen MK Neergaard MA Jensen AB Bro F Guldin MB. Psychological distress, health, and socio-economic factors in caregivers of terminally ill patients: a nationwide population-based cohort study. Support Care Cancer. (2016) 24:3057–67. 10.1007/s00520-016-3120-7 26887588

[62] Ganapathy V Graham GD DiBonaventura MD Gillard PJ Goren A Zorowitz RD. Caregiver burden, productivity loss, and indirect costs associated with caring for patients with poststroke spasticity. Clin Interv Aging. (2015) 10:1793–802. 10.2147/CIA.S91123 26609225 PMC4644168

[63] Byun E Evans LK. Concept analysis of burden in caregivers of stroke survivors during the early poststroke period. Clin Nurs Res. (2015) 24:468–86. 10.1177/1054773814537060 24913926

[64] Rubbens E De Clerck L Swinnen E Effectiveness of interventions to decrease the physical and mental burden and strain of informal caregivers of stroke patients: a systematic review. In: Ibáñez J González-Vargas Azorin JM Akay M Pons JL editors. Converging Clinical and Engineering Research on Neurorehabilitation II. Segovia: Springer (2017). p. 299–303.

[65] World Health Organization Cancer Pain Relief and Palliative Care: Report of a WHO Expert Committee. Geneva (1990). 1702248

[66] Morrison RS Meier DE. Clinical practice: palliative care. N Engl J Med. (2004) 350:2582–90. 10.1056/NEJMcp035232 15201415

[67] Oliver D de Visser M Voltz R. Palliative care in neurology. Lancet Neurol. (2017) 16:868. 10.1016/S1474-4422(17)30321-6 29029843

[68] Adil MM Zweifler R Abstract WMP56: increase in rate of utilization of withdrawal of care in acute ischemic stroke patients in USA. Stroke. (2017) 48(Suppl. 1). Available online at: http://n.neurology.org/content/88/16_Supplement/P1.209.short

[69] Lynn J. Serving patients who may die soon and their families: the role of hospice and other services. JAMA. (2001) 285:925–32. 10.1001/jama.285.7.925 11180736

[70] Murray SA Boyd K Sheikh A. Palliative care in chronic illness. BMJ. (2005) 330:611–2. 10.1136/bmj.330.7492.611 15774965 PMC554893

[71] Moss AH Lunney JR Culp S Auber M Kurian S Rogers J. Prognostic significance of the “surprise” question in cancer patients. J Palliat Med. (2010) 13:837–40. 10.1089/jpm.2010.0018 20636154

[72] Cadilhac DA Moss KM Price CJ Lannin NA Lim JY Anderson CS. Pathways to enhancing the quality of stroke care through national data monitoring systems for hospitals. Med J Aust. (2013) 199:650–1. 10.5694/mja12.11821 24237082

[73] Kwan J Sandercock P. In-hospital care pathways for stroke: a Cochrane systematic review. Stroke. (2003) 34:587–8. 10.1161/01.STR.0000054673.28010.1B 12574581

[74] Phillips JL Halcomb EJ Davidson PM. End-of-life care pathways in acute and hospice care: an integrative review. J Pain Sympt Manage. (2011) 41:940–55. 10.1016/j.jpainsymman.2010.07.020 21398083

[75] Radbruch L Loick G Sabatowski R Elsner F. MIDOS–an electronic database for the palliative care unit. Schmerz. (2000) 14:257–63. 10.1007/s004820070032 12800033

[76] Steigleder TMC Huber D Ostgathe C Schramm A Botulinumtoxin in der Palliativmedizin – Entwicklung eines Screening-Instrumentes zur Identifikation von Patienten, die von Botulinumtoxintherapie profitieren. In: 11. Kongress der Deutschen Gesellschaft für Palliativmedizin. Leipzig (2016).

[77] Steigleder T Möbius C Huber D Ostgathe C Botulinumtoxin in der Palliativmedizin–Entwicklung eines Screening-Instrumentes zur Identifikation von Patienten, die von Botulinumtoxintherapie profitieren. Z Palliativmed. (2016) 17:1–59. 10.1055/s-0036-1594036

[78] Furlan AD Sandoval JA Mailis-Gagnon A Tunks E. Opioids for chronic noncancer pain: a meta-analysis of effectiveness and side effects. Can Med Assoc J. (2006) 174:1589–94. 10.1503/cmaj.051528 16717269 PMC1459894

[79] Shaw LC Price CIM van Wijck FMJ Shackley P Steen N Barnes MP. Botulinum Toxin for the Upper Limb after Stroke (BoTULS) Trial: effect on impairment, activity limitation, and pain. Stroke. (2011) 42:1371–9. 10.1161/STROKEAHA.110.582197 21415398

[80] Dionne-Odom JN Azuero A Lyons KD Hull JG Tosteson T Li Z. Benefits of early versus delayed palliative care to informal family caregivers of patients with advanced cancer: outcomes from the ENABLE III randomized controlled trial. J Clin Oncol. (2015) 33:1446. 10.1200/JCO.2014.58.7824 25800762 PMC4404423

[81] Longacre ML Applebaum AJ Buzaglo JS Miller MF Golant M Rowland JH. Reducing informal caregiver burden in cancer: evidence-based programs in practice. Transl Behav Med. (2018) 8:145–55. 10.1093/tbm/ibx028 29385550 PMC6257028

[82] Spatuzzi R Giulietti MV Ricciuti M Merico F Meloni C Fabbietti P. Quality of life and burden in family caregivers of patients with advanced cancer in active treatment settings and hospice care: a comparative study. Death Stud. (2017) 41:276–83. 10.1080/07481187.2016.1273277 27982741

[83] Kendall M Is palliative care appropriate for all people with major stroke? A multi-centre, mixed-method, longitudinal study of outcomes and experiences. Can Med Assoc J. (2017). 10.1503/cmaj.170604 PMC5837872 29507155

